# Degradation of SAMHD1 Restriction Factor Through Cullin-Ring E3 Ligase Complexes During Human Cytomegalovirus Infection

**DOI:** 10.3389/fcimb.2020.00391

**Published:** 2020-07-30

**Authors:** Seokhwan Hyeon, Myoung Kyu Lee, Young-Eui Kim, Gwang Myeong Lee, Jin-Hyun Ahn

**Affiliations:** Department of Microbiology, Sungkyunkwan University School of Medicine, Suwon-si, South Korea

**Keywords:** HCMV, SAMHD1, restriction factor, degradation, CRL

## Abstract

Sterile alpha motif (SAM) and histidine-aspartate (HD) domain-containing protein 1 (SAMHD1) acts as a restriction factor for several RNA and DNA viruses by limiting the intracellular pool of deoxynucleoside triphosphates. Here, we investigated the regulation of SAMHD1 expression during human cytomegalovirus (HCMV) infection. SAMHD1 knockdown using shRNA increased the activity of the viral UL99 late gene promoter in human fibroblasts by 7- to 9-fold, confirming its anti-HCMV activity. We also found that the level of SAMHD1 was initially increased by HCMV infection but decreased partly at the protein level at late stages of infection. SAMHD1 loss was not observed with UV-inactivated virus and required viral DNA replication. This reduction of SAMHD1 was effectively blocked by MLN4924, an inhibitor of the Cullin-RING-E3 ligase (CRL) complexes, but not by bafilomycin A1, an inhibitor of vacuolar-type H^+^-ATPase. Indirect immunofluorescence assays further supported the CRL-mediated SAMHD1 loss at late stages of virus infection. Knockdown of CUL2 and to a lesser extent CUL1 using siRNA stabilized SAMHD1 in normal fibroblasts and inhibited SAMHD1 loss during virus infection. Altogether, our results demonstrate that SAMHD1 inhibits the growth of HCMV, but HCMV causes degradation of SAMHD1 at late stages of viral infection through the CRL complexes.

## Introduction

Cellular restriction factors play an important role in limiting the growth of a virus in a certain cell type. Sterile alpha motif (SAM) and histidine-aspartate (HD) domain-containing protein 1 (SAMHD1) was initially identified as a restriction factor that inhibits HIV-1 infection and shown to be counteracted by the viral Vpx protein through proteasomal degradation (Berger et al., 2011; Hrecka et al., 2011; Laguette et al., 2011; Baldauf et al., 2012). The antiviral activity of SAMHD1 is associated with reduction of deoxynucleoside triphosphates (dNTP) pools and its dNTP triphosphohydrolase (dNTPase) activity (Goldstone et al., 2011; Powell et al., 2011; Baldauf et al., 2012; Lahouassa et al., 2012; Franzolin et al., 2013; Hollenbaugh et al., 2014). Involvement of RNase activity in the restriction function of SAMHD1 has also been proposed (Beloglazova et al., 2013; Ryoo et al., 2014), but its importance remains unclear (Seamon et al., 2015; Antonucci et al., 2016).

In addition to retroviruses, SAMHD1 has been shown to restrict the growth of other RNA or DNA viruses, such as human T cell leukemia virus type 1 (Sze et al., 2013), hepatitis B virus (Chen et al., 2014; Jeong et al., 2016; Sommer et al., 2016), vaccinia virus (Hollenbaugh et al., 2013), herpes simplex virus type-1 (Hollenbaugh et al., 2013; Kim et al., 2013; Badia et al., 2016), murine cytomegalovirus (MCMV) (Deutschmann et al., 2019), and human cytomegalovirus (HCMV) (Businger et al., 2019; Kim et al., 2019). HCMV belongs to the β-herpesvirus subfamily and contains a 235-kb double-stranded DNA genome. Although HCMV infection in healthy people is generally asymptomatic, congenital infection in pregnant women or reactivation or re-infection in immunocompromised people often causes disease complications (Mocarski et al., 2013). Recent studies reported that SAMHD1 inhibits HCMV growth by lowering viral immediate-early gene expression through modulation of NF-κB level or DNA replication in primary human fibroblasts (HFs) and monocyte-derived macrophages (MDMs) (Businger et al., 2019; Kim et al., 2019). SAMHD1 is phosphorylated at T592 mainly by cyclin-dependent kinases (CDKs), which blocks its antiviral activity (Cribier et al., 2013; White et al., 2013; Pauls et al., 2014; St Gelais et al., 2014; Yan et al., 2015). Expression of SAMHD1 and its phosphorylation are initially increased during HCMV infection (Kim et al., 2019). Of note, the HCMV or MCMV-encoded kinases phosphorylate SAMHD1, counteracting the antiviral activity of SAMHD1 (Businger et al., 2019; Deutschmann et al., 2019; Kim et al., 2019).

The steady-state expression of SAMHD1 is downregulated in MDMs at both transcriptional and post-transcriptional levels (Businger et al., 2019). Although this suggests that HCMV might have evolved several strategies to finely regulate SAMHD1 level during productive infection, the underlying mechanisms have not been studied. In this study, we investigated how SAMHD1 expression is regulated during productive infection in primary HF cells. We found that SAMHD1 protein level is initially upregulated after infection, but it is downregulated at late stages of infection. Loss of SAMHD1 protein was found to require viral DNA replication and involve the activity of Cullin-RING-E3 ligase (CRL) complexes.

## Materials and Methods

### Cells, Viruses, and Chemicals

Primary human foreskin fibroblast (HF) cells (ATCC® SCRC-1041™) and human embryonic kidney 293T cells were grown in Dulbecco's modified Eagle's medium (DMEM) supplemented with 10% fetal bovine serum in a 5% CO_2_ humidified incubator at 37°C. The cell culture medium contained 100 units/ml of penicillin and 100 μg/ml of streptomycin. HF cells of passage number 15–20 were used. Stocks for HCMV (Towne strain) were prepared in HF cells as described previously (Ahn et al., 1998). Recombinant HCMV (Toledo strain) was produced from the Toledo-bacmid gifted from Hua Zhu (UMDNJ-New Jersey Medical School) (Kwon et al., 2017). A clinical isolate JHC was described previously (Yi et al., 2009). Recombinant HCMVs harboring the polymerase (UL54)-luciferase or the pp28 (UL99)-luciferase reporter construct were also described previously and provided by Gary S. Hayward (Johns Hopkins Medicine) (Ahn and Hayward, 2000; He et al., 2011). UV-inactivated viruses were produced by irradiating the virus stock with UV light three times at 0.72 J/cm^2^ using a CL-1000 cross-linker (UVP). MG132, MLN4924, phosphonoacetic acid (PAA), and bafilomycin A1 were purchased from Calbiochem, Bostonbiochem, Sigma-Aldrich, and Glentham, respectively.

### Plasmids, siRNA, and Transfection

pENTR plasmid (Invitrogen) containing the SAMHD1 cDNA was provided by Eui Tae Kim (University of Pennsylvania). Expression plasmid for HA-SAMHD1 was produced by transferring the DNA to pSG5 (Green et al., 1988)-based destination vector using LR Clonase (Invitrogen). Sequences for control siRNA and siRNAs for CUL1, CUL2, CUL3, and CUL4 and their specificity were previously described (Cukras et al., 2014). These siRNAs were purchased from GenePharma.

#### Lentiviral Vectors for Short Hairpin RNA (shRNA) Expression

Lentiviral vector pLKO.1-TRC control expressing a non-hairpin control RNA (shC) was purchased from Addgene. Lentiviral vectors (on a pLKO.1 background) expressing shRNAs against SAMHD1 [shSAMHD1-#1 and -#2, which correspond to TRCN0000343807 (5′-CCCTGAAGAAGATATTTGCTT-3′) and TRCN0000343808 (5′- GCCATCATCTTGGAATCCAAA−3′), respectively] were described previously (Kim et al., 2019) and provided by Eui Tae Kim. For lentivirus production, 293T cells were co-transfected with lentiviral vectors and plasmids pCMV-DR8.91 expressing the human immunodeficiency virus (HIV) gag-pol, tat, and rev proteins and pMD-G expressing the vesicular stomatitis virus (VSV) envelope G protein (Pham et al., 2013). The culture media containing lentiviruses were collected at 2 days after transfection and stored at −70°C until use. HF cells were transduced with lentiviruses in the presence of polybrene (7.5 μg/ml) and selected with puromycin (2 μg/ml). The selected cells were maintained in a medium containing puromycin (0.5 μg/ml).

#### Antibodies

Anti-SAMHD1 mouse monoclonal antibody (MAb) (OTI1A1) was obtained from Origene. Anti-HA rat MAb (3F10) conjugated with horseradish peroxidase (HRP) was purchased from Roche. Anti-IE1/IE2 mouse MAb (810R) was obtained from Chemicon. Mouse MAbs for UL44 (pp52) (10D8) and UL99 (pp28) (CH19) were purchased from Virusys. Mouse MAbs for p21^CIP1^ (CP26, CP74) and β-actin (5B7) were purchased from EnoGene and Upstate, respectively. Anti-IE1 and anti-UL112-113 rabbit polyclonal antibodies (PAb) were previously described (Ahn et al., 1999; Kim et al., 2014).

### Indirect Immunofluorescence Assay (IFA)

Cells were fixed in 4% paraformaldehyde for 5 min at room temperature and permeabilized in 0.2% triton X-100 in PBS at 4°C for 20 min. Cells were first incubated with human γ-globulin (Sigma) (2 mg per ml) for 1 h at 37°C to block non-specific binding of antibodies to HCMV-induced Fc receptors (Antonsson and Johansson, 2001). Cells were incubated with primary antibodies [anti-IE1 rabbit PAb (1:2000) and anti-SAMHD1 mouse MAb (1:200)] in PBS at 37°C for 1 h and then with secondary antibodies [fluorescein isothiocyanate (FITC)-labeled anti-rabbit IgG or rhodamine/Red X-coupled anti-mouse IgG] (Jackson ImmunoResearch Inc.) at 37°C for 1 h. Hoechst stain was used to stain cell nuclei. The slides were examined and photographed with a Carl Zeiss LSM710Meta confocal microscope system.

#### Immunoblot Analysis

Cells were washed with phosphate-buffered saline (PBS), and total cell lysates were prepared by boiling the cell pellets in sodium dodecyl sulfate (SDS) loading buffer. Equal amounts of the clarified cell extracts were separated on an SDS-polyacrylamide gel and electroblotted onto nitrocellulose membranes. The blots were blocked by incubation in PBS plus 0.1% Tween 20 (PBST) containing 5% non-fat dry milk for 1 h at room temperature. The blots were washed with PBST three times and incubated with the appropriate antibodies in PBST for 1 h at room temperature. After three 5-min washes with PBST, the blots were incubated with HRP-conjugated goat anti-mouse IgG or anti-rabbit IgG (Amersham) for 1 h at room temperature. The blots were then washed three times with PBST, and the protein bands were visualized with an enhanced chemiluminescence system (Amersham). The relative protein levels in immunoblots were quantitated using ImageJ (NIH).

#### Reverse Transcription (RT)-PCR

Total RNA was isolated from 2 × 10^5^ cells using TriZOL reagent (Invitrogen). cDNAs were synthesized using the random hexamer primers in the SuperScript III system (Invitrogen). Quantitative RT-PCR (qRT-PCR) was performed using the Power SYBR Green PCR Master Mix and QuantStudio Real-Time PCR System. The following primers were used: SAMHD1 forward 5′-CGAGATGTTCTCTGTGTTCA-3′ and reverse 5′- CGTCCATCAAACATGTGAGA-3′; β-actin forward 5′-AGCGGGAAATCGTGCGTG-3′ and reverse 5′-CAGGGTACATGGTGGTGCC-3′.

#### Infectious Center Assay

The infectious units of viral stocks were determined by infectious center assays. Diluted samples were used to inoculate a monolayer of HF cells (1 × 10^5^) in a 24-well plate. At 24 h post-infection, cells were fixed with 500 μl of cold methanol for 10 min. Cells were then washed three times in PBS and incubated with anti-IE1 rabbit polyclonal antibody in PBS at 37°C for 1 h, followed by incubation with phosphatase-conjugated anti-rabbit IgG antibody in PBS at 37°C for 1 h. The cells were then gently washed in PBS and treated with 200 μl of developing solution (nitroblue tetrazolium/5-bromo-4-chloro-3-indolylphosphate) at room temperature for 1 h according to the manufacturer's instructions. The IE1-positive cells were counted in at least three separate fields per well under a light microscope (200X magnification).

#### Luciferase Reporter Assay

Cells were washed with PBS and lysed by three freeze-thaw steps in 200 μl of 0.25 M Tris-HCl (pH 7.9) plus 1 mM dithiothreitol. After clarification of cell lysates in a microcentrifuge, 30 μl of extracts were incubated with 350 μl of reaction buffer A (25 mM glycyl-glycine [pH 7.8], 15 mM ATP, and 4 mM EGTA) and then mixed with 100 μl of 0.25 mM luciferin (Sigma-Aldrich) in reaction buffer A. A TD-20/20 luminometer (Turner Designs) was used for a 10-s assay of the photons produced (measured in relative light units).

#### Statistical Analysis

Statistical significance between samples was determined using Student's *t*-test. *P* < 0.05 was considered to indicate statistical significance.

## Results

### SAMHD1 Restricts HCMV Late Gene Expression in Human Fibroblasts

**Figure 1:**
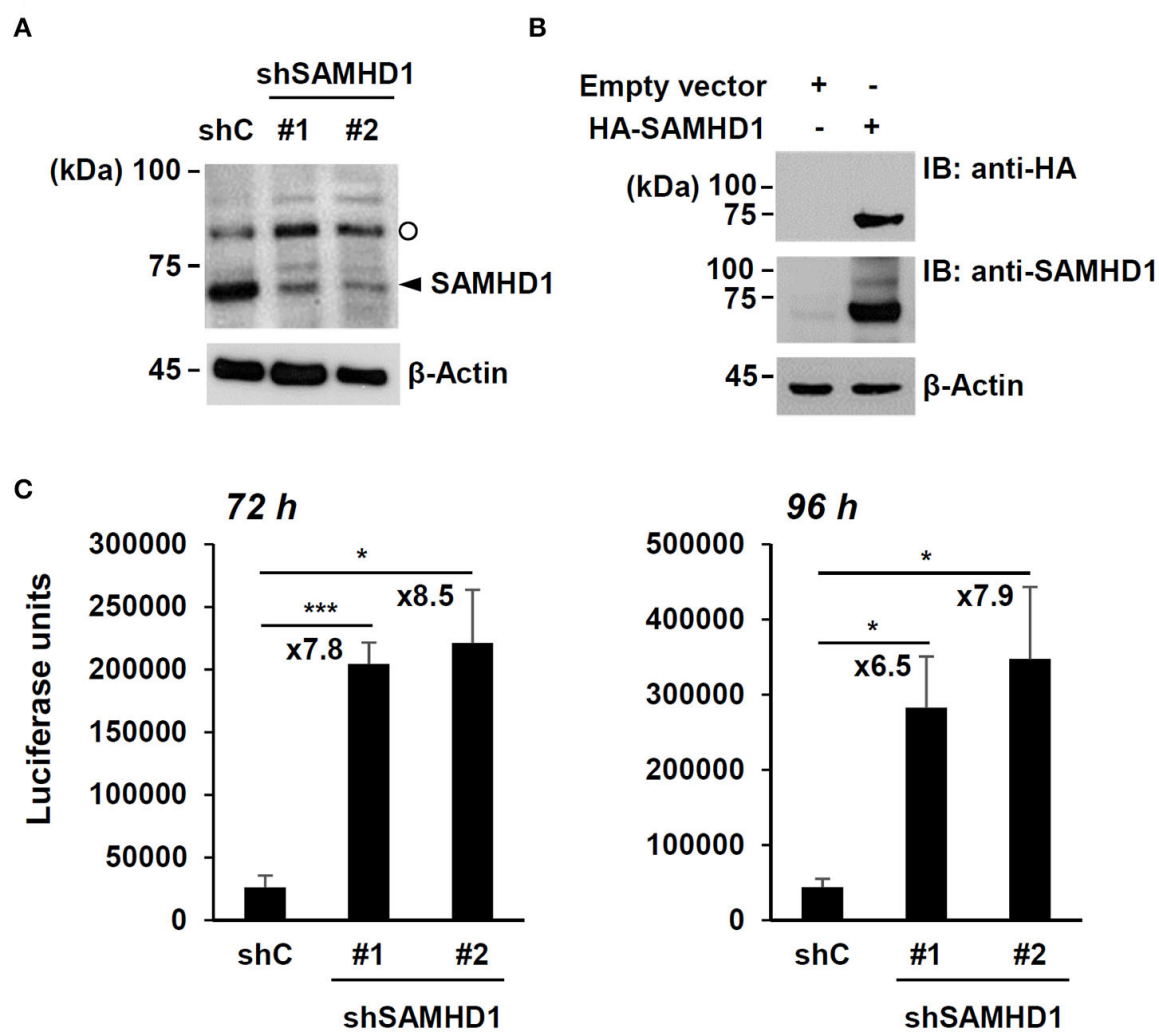
Effect of SAMHD1 depletion on HCMV growth in HF cells. **(A)** HF cells were transduced by lentiviral vectors expressing control shRNA (shC) or shRNAs for SAMHD1 (shSAMHD1-#1 and shSAMHD1-#2). At 24 h after transduction, total cell lysates were prepared and subjected to SDS-10% PAGE, followed by immunoblotting with anti-SAMHD1 antibody. The level of β-actin is shown as a loading control. The band position corresponding to SAMHD1 is indicated with an arrowhead. Non-specific bands are indicated as an open circle. **(B)** 293T cells were transfected with empty vector or plasmid expressing HA-SAMHD1 for 48 h. Total cell lysates were prepared and immunoblotted with anti-HA or anti-SAMHD1 antibodies. **(C)** HF cells expressing control shRNA (shC) or shSAMHD1 were infected with recombinant HCMV (Towne) containing the UL99-luciferase reporter gene at an MOI of 3 (infectious unit per ml) for 72 and 96 h. The luciferase activity within the cell lysates was measured. Data are shown as mean values with standard errors from three independent assays. Values of *P < 0.05 and ***P < 0.001 are indicated.

To confirm the anti-HCMV activity of SAMHD1, SAMHD1-depleted HF cells were produced by transduction with lentiviral vectors expressing shRNAs. The expression of endogenous SAMHD1 was reduced by two shRNAs (shSAMHD1-#1 and -#2) (Figure 1A). Immunoblotting of cell lysates with anti-SAMHD1 antibody detected another slowly migrating band. However, this band appeared to be non-specific because it was not reduced in cells expressing SAMHD1 shRNA (Figure 1A) and not detected in HA-SAMHD1-transfected 293T cells by immunoblotting with anti-HA antibody (Figure 1B).

We then infected control and SAMHD1-knockdown cells with recombinant HCMV (Towne strain) containing the UL99-luciferase reporter gene, in which luciferase gene expression is driven by the viral UL99 late gene promoter, at a multiplicity of infection (MOI) of 3 for 72 h or 96 h and measured the amounts of luciferase produced in cell lysates. The luciferase units were about 7- to 9-fold higher in shSAMHD1-#1 and shSAMHD1-#2 cells than in control (shC) cells (Figure 1C). These results confirmed that SAMHD1 restricts HCMV growth.

### Regulation of SAMHD1 Expression During HCMV Infection

**Figure 2:**
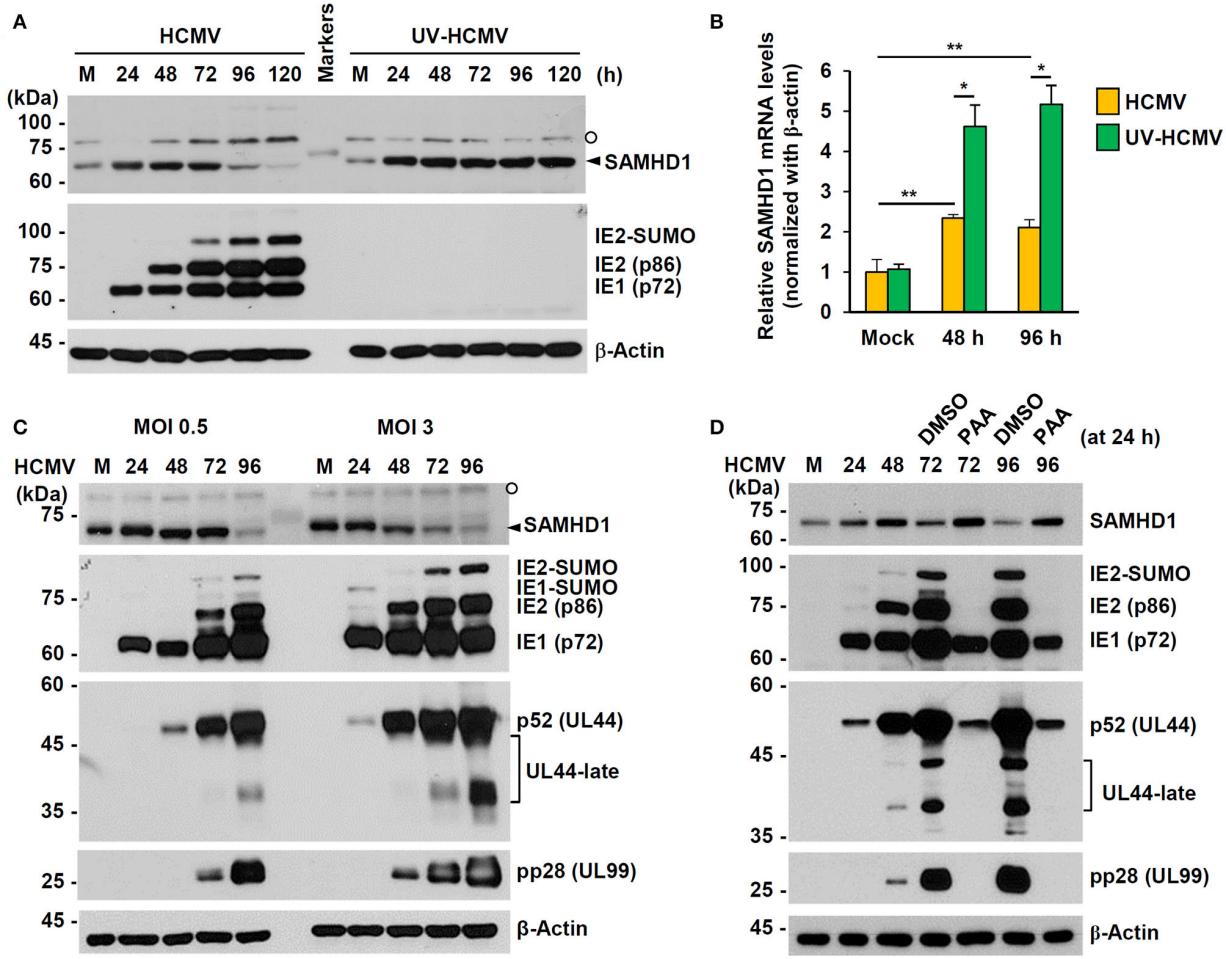
Expression patterns of SAMHD1 during HCMV infection in HF cells. **(A)** Non-synchronized sub-confluent HF cells in six-well plates were mock-infected (M) or infected with intact HCMV (Towne) or UV-inactivated virus (UV-HCMV) at an MOI of 1. Total cell lysates were prepared at the indicated time points, subjected to SDS-8% PAGE, and immunoblotted with antibodies for SAMHD1, IE1/IE2, and β-actin. SAMHD1 bands are indicated with an arrowhead and non-specific bands are indicated as an open circle. **(B)** HF cells were infected as in **(A)**. Total mRNAs were prepared at the indicated time points, and the levels of SAMHD1 and β-actin transcripts were measured by qRT-PCR. The relative SAMHD1 mRNA level is normalized with the level of β-actin. Results shown are mean values and standard errors of three independent experiments. Values of *P < 0.05 and **P < 0.01 are indicated. **(C)** HF cells in six-well plates were mock-infected or infected with HCMV (Towne) at an MOI of 0, 5, or 3, and harvested at the indicated time points. Total cell lysates were prepared and subjected to SDS-8% PAGE, followed by immunoblotting with antibodies for SAMHD1, IE1/IE2, p52 (UL44), pp28 (UL99), and β-actin. **(D)** HF cells in six-well plates were mock-infected or infected with HCMV (Towne) at an MOI of 1 and harvested at the indicated time points. For inhibitor treatment, cells were treated with DMSO or phosphonoacetic acid (PAA) (200 μg/ml; Sigma-Aldrich) at 24 h post infection. Total cell lysates were prepared and subjected to SDS-8% PAGE, followed by immunoblotting with antibodies for SAMHD1, IE1/IE2, p52 (UL44), pp28 (UL99), and β-actin.

We investigated how SAMHD1 expression is regulated during HCMV infection. First, HF cells were infected with HCMV (Towne) or UV-inactivated virus (UV-HCMV) at an MOI of 1, and the amounts of SAMHD1 protein in infected cells were analyzed at different time points by immunoblot assays. We found that the amounts of SAMHD1 initially increased up to 48 h or 72 h but decreased after that, and that loss of SAMHD1 at late stages of infection was not observed with UV-HCMV (Figure 2A). The lack of SAMHD1 loss with UV-HCMV suggests that the downregulation requires viral gene expression. In similar virus-infected cells, we also measured SAMHD1 mRNA levels by qRT-PCR at 48 and 96 h after infection. Most of cells were infected with intact HCMV at these time points. We found that SAMHD1 mRNA levels were increased at 48 or 96 h with both HCMV and UV-HCMV, but the levels in HCMV-infected cells were lower than those in UV-HCMV-infected cells (Figure 2B). This suggests that, consistent with the observation in MDMs (Businger et al., 2019), SAMHD1 induction is at least partly downregulated by viral products at the transcriptional level in HF cells. Notably, SAMHD1 mRNA levels at 48 and 96 h in HCMV-infected cells were not significantly changed (Figure 2B), indicating that SAMHD1 protein loss at late stages of HCMV infection shown in Figure 2A also involves post-transcriptional regulation.

When we compared SAMHD1 protein levels at different MOIs (0.5 and 3), SAMHD1 loss occurred at 96 h at an MOI of 0.5, while it was observed as early as 48 h at an MOI of 3, indicating that when SAMHD1 loss is observed after HCMV infection depends on MOIs (Figure 2C). SAMHD1 loss generally correlated with the high level expression of viral late proteins, such as the SUMO-modified form of viral immediate-early (IE) 2 (IE2-SUMO) and UL99-encoded pp28, indicating that the overall reduction of SAMHD1 level is evident at late stages of infection. The expression of viral late genes requires the initiation of viral DNA replication. Therefore, we examined the effect of phosphonoacetic acid (PAA), an inhibitor of viral DNA polymerase, on loss of SAMHD1. Consistent with our previous assay, SAMHD1 level was reduced at 72 and 96 h after infection at an MOI of 1; however, it was almost completely blocked when cells were treated with PAA at 24 h after infection, demonstrating that SAMHD1 loss requires viral DNA replication and/or viral late gene expression (Figure 2D).

### SAMHD1 Loss Is Dependent on CRL Complexes

**Figure 3:**
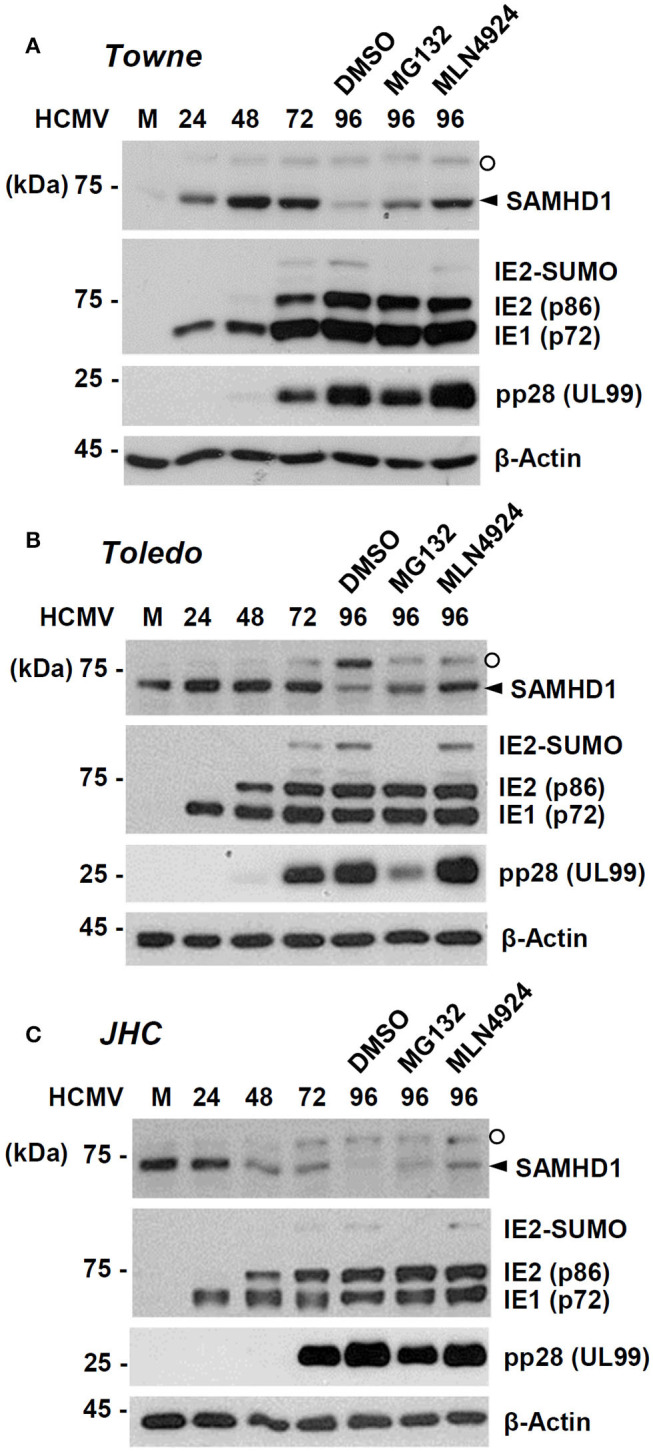
Effects of proteasome or CRL inhibitors on SAMHD1 accumulation in HCMV-infected cells. HF cells in six-well plates were mock-infected or infected with different HCMV strains [Towne **(A)**, Toledo **(B)**, or JHC **(C)**] at an MOI of 1. Cells were harvested at the indicated time points. For inhibitor treatment, cells were treated with DMSO, MG132 (5 μM), or MLN4924 (1 μM) at 72 h post infection and incubated for another 24 h. Total cell lysates were prepared, subjected to SDS-8% PAGE, and immunoblotted with antibodies for SAMHD1, IE1/IE2, pp28 (UL99), and β-actin. The SAMHD1 and non-specific bands are indicated as arrowheads and open circles, respectively.

We further investigated how SAMHD1 loss occurs at the post-transcriptional level. HF cells were infected with HCMV (Towne) at an MOI of 1. At 72 h after infection, cells were treated with DMSO, proteasome inhibitor MG132, or MLN4924, a Nedd8-activating enzyme (NAE) inhibitor that blocks formation of the CRL complex. The level of SAMHD1 was measured at different time points with or without inhibitors by immunoblot assays. We found that loss of SAMHD1 was only partially inhibited by MG132 but very effectively blocked by MLN4924, suggesting a critical role of the CRL complexes in loss of SAMHD1 (Figure 3A). We observed a similar loss of SAMHD1 and a similar effect of MLN4924 during infection of Toledo and JHC strains, suggesting that this SAMHD1 regulation is well-conserved among HCMV strains, and that the UL/b′ region, which is absent in the Towne strain, is not required for this regulation (Figures 3B,C). Compared to Towne or Toledo-infected cells, JHC-infected cells showed a decrease of SAMHD1 level from 48 h after infection, although the complete loss of SAMHD1 was observed at 96 h after infection, as in other laboratory strains. We previously found that infection with the JHC stain led to a higher induction of COX-2 expression compared to infection with the laboratory strains (Yi et al., 2009). We infer that different cellular responses to JHC infection may be related to this difference. Notably, MG132 treatment reduced accumulation of viral late proteins, such as the SUMO-modified form of IE2 and pp28. Therefore, it was unclear whether this effect of MG132 was caused by the inactivation of proteasomes or by a decrease in viral late gene expression. However, MLN4924 did not affect late gene expression but almost completely blocked the virus-induced SAMHD1 loss. These results with MLN4924 indicate that loss of SAMHD1 largely occurs at the protein level and is dependent on formation of CRL complexes.

**Figure 4:**
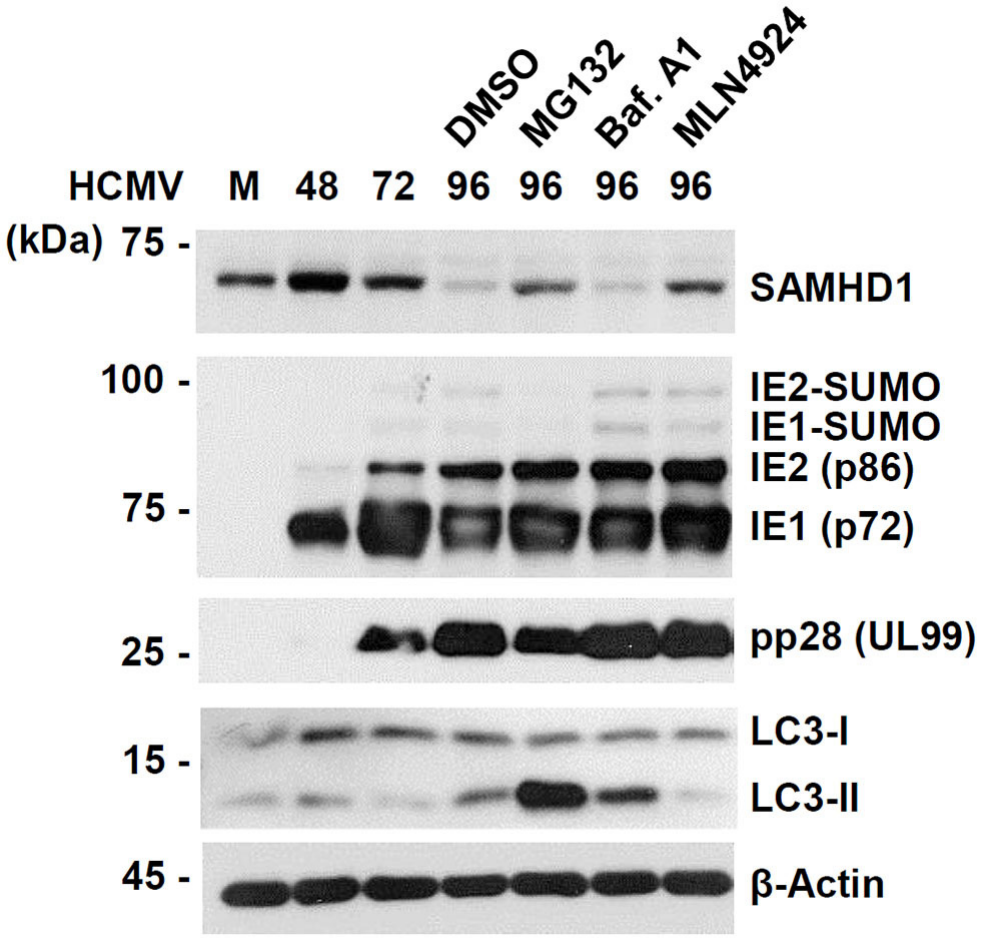
Comparative analysis of SAMHD1 loss using inhibitors. HF cells in six-well plates were mock-infected or infected with different HCMV (Towne) at an MOI of 1. Cells were harvested at the indicated time points. For inhibitor treatment, cells were treated with DMSO, MG132 (5 μM), bafilomycin A1 (100 nM), or MLN4924 (1 μM) at 72 h post infection and incubated for another 24 h. Total cell lysates were prepared, subjected to SDS-8% PAGE, and immunoblotted with antibodies for SAMHD1, IE1/IE2, pp28 (UL99), LC3, and β-actin.

To further investigate the mechanism by which SAMHD1 is lost during HCMV infection, we compared the effects of MG132, MLN4924, and bafilomycin A1, a specific and potent inhibitor of vacuolar-type H+-ATPases that blocks lysosomal degradation, on CRL-mediated SAMHD1 loss. We found that treatment of MG132 or MLN4924 at 72 h after infection partly inhibited SAMHD1 loss at 96 h as above, however, bafilomycin A1 did not inhibit SAMHD1 loss at all, suggesting that the virus-induced SAMHD1 loss occurs through the non-lysosomal pathways (Figure 4). As a control, bafilomycin A1 increased the lipid-modified form of LC3 (LC3-II), confirming its activity. Interestingly, MG132 also markedly increased LC3-II level in HCMV-infected cells.

### Alteration of SAMHD1 Localization at Late Stages of HCMV Infection

**Figure 5:**
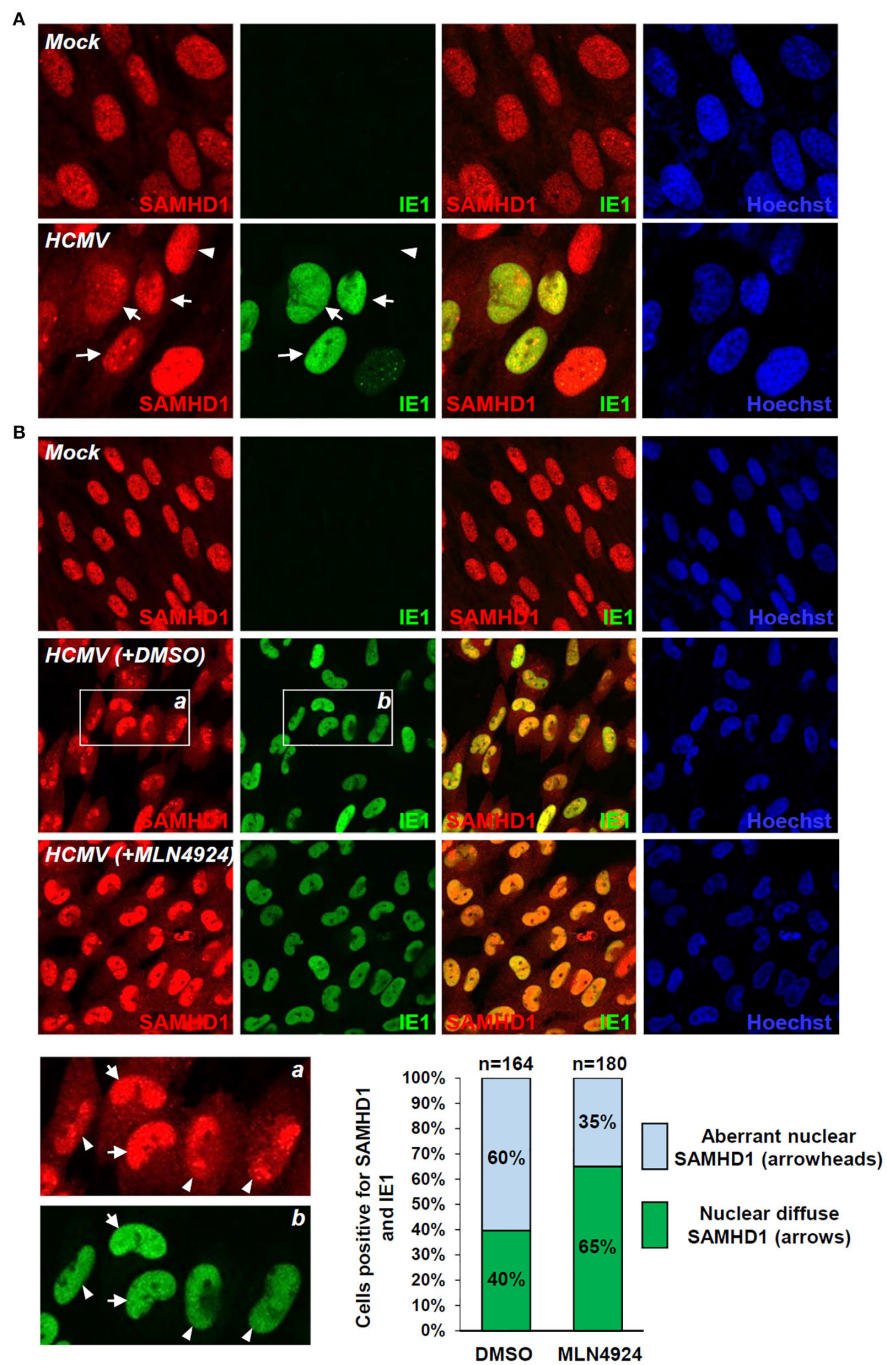
IFA demonstrating the CRL-mediated SAMHD1 loss during HCMV infection. **(A)** HF cells in chamber slides were mock infected or infected with HCMV (Towne) at an MOI of 1 for 72 h and double-label confocal IFA was performed with anti-SAMHD1 and anti-IE1 antibodies. FITC-labeled anti-rabbit IgG and rhodamine/Red X-coupled anti-mouse IgG were used for visualization. Hoechst stain was used to stain cell nuclei. Three single-labeled images and a merged imaged for SAMHD1 and IE1 are shown. Some virus-infected (arrows) and uninfected (arrowheads) cells were indicated. **(B)** HF cells were mock-infected or infected with HCMV for 96 h with DMSO or MLN4924 treatment for 24 h prior to cell fixation and double-label confocal IFA was performed as in **(A)**. Inserts (a and b) are enlarged to indicate cells showing nuclear diffuse distribution of SAMHD1 (arrows) or aberrant nuclear distribution of SAMHD1 (arrowheads). Percentages (%) of cells showing different SAMHD1 distribution patterns in DMSO or MLN4924-treated cells are shown as graphs. The total cell numbers (n) counted are indicated.

SAMHD1 loss during the late stages of HCMV infection was further investigated by IFA. SAMHD1 was predominantly localized in the nuclei of mock-infected HF cells. When cells were infected with HCMV at an MOI of 1 for 72 h, some virus-infected (IE1-positive) cells showed a decrease of nuclear SAMHD1 and an increase of cytoplasmic SAMHD1 (Figure 5A, arrows), compared to uninfected (IE1-negative) cells showing only nuclear SAMHD1 (Figure 5A, arrowheads). We also treated cells with DMSO or MLN4924 for 24 h prior to cell fixation at 96 h after infection. When treated with DMSO, about 60% of virus-infected cells showed aberrant nuclear distribution of SAMHD1 (Figure 5B, see arrowheads), while 40% of infected cells showed nuclear diffuse distribution of SAMHD1 (Figure 5B, arrows). However, in MLN4924-treated cells, the nuclear diffuse SAMHD1 levels were higher compared to those in DMSO-treated cells and cells showing aberrant nuclear distribution of SAMHD1 was reduced to 35% (Figure 5B). These IFA results demonstrate that the nuclear SAMHD1 level is reduced and its distribution is changed at late times of HCMV infection in a manner partly dependent on the CRL activity, supporting the finding that SAMHD1 is lost in a CRL-dependent manner during the late stages of HCMV infection.

**Figure 6:**
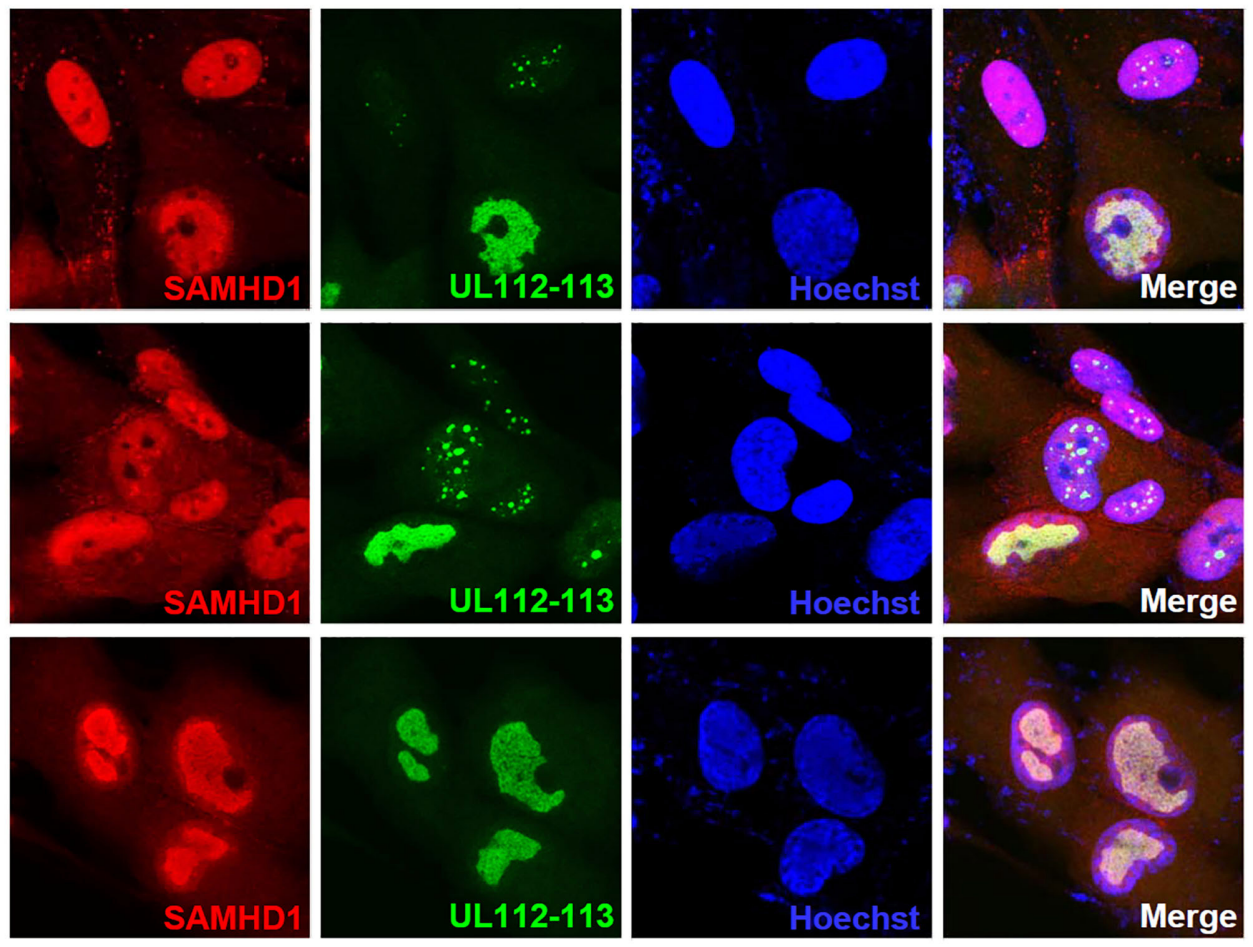
Localization of SAMHD1 in viral replication compartments. HF cells in chamber slides were infected with HCMV (Towne) at an MOI of 1 for 90 h and double-label confocal IFA was performed with anti-SAMHD1 and anti-UL112-113 p84 antibodies. FITC-labeled anti-rabbit IgG and rhodamine/Red X-coupled anti-mouse IgG were used for visualization. Hoechst stain was used to stain cell nuclei. Three single-labeled images and a merged image for SAMHD1 and UL112-113 are shown. The top and middle images were selected to include some cells showing UL112-113 foci, while the bottom images show cells with fully grown viral replication compartments.

SAMHD1 has been shown to act at stalled replication forks to allow the forks to restart replication (Coquel et al., 2018). Since the aberrant nuclear localization pattern of SAMHD1 found at late times of infection resembles viral replication compartments, we tested whether SAMHD1 is associated with these sites. The double-label IFA results using the antibody specific for UL112-113-encoded p84 that accumulates at viral replication compartments (Kim and Ahn, 2010; Kim et al., 2015) showed that SAMHD1 is partly colocalized with UL112-113 p84 in viral replication compartments (Figure 6).

### Regulation of SAMHD1 Stability by CUL1 and CUL2

**Figure 7:**
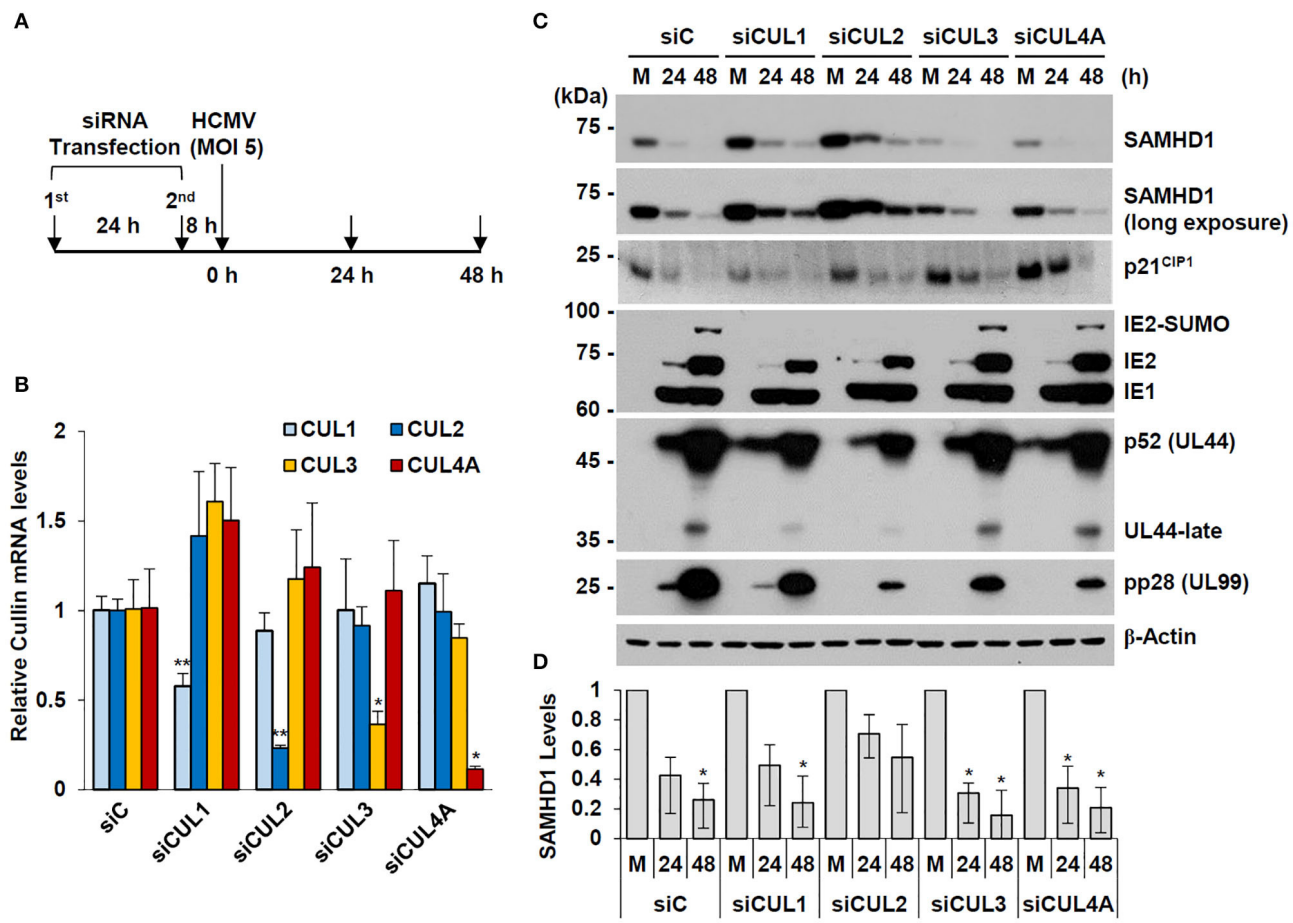
Effect of Cullin depletion on HCMV-induced SAMHD1 loss. **(A)** Schematic of the experimental workflow. HF cells in six-well plates were transfected with siRNA twice during 24 h and then infected with HCMV (Towne) at an MOI of 5. **(B)** HF cells were transfected with control siRNA or siRNAs for CUL1, CUL2, CUL3, or CUL4, and their mRNA levels were measured by qRT-PCR. The relative SAMHD1 mRNA level is normalized with the level of β-actin. Results shown are mean values and standard errors of three independent experiments. Values of *P < 0.05 and **P < 0.01 are indicated. **(C)** siRNA-transfected HF cells were mock-infected (M) or infected with HCMV as described in **(A)**. Total cell lysates were prepared at 24 and 48 h post infection and immunoblotted with antibodies for SAMHD1, p21^CIP1^, IE1/IE2, p52 (UL44), pp28 (UL99), and β-actin. **(D)** The relative SAMHD1 protein level normalized with that of β-actin is shown on the graph. Data are shown as mean values with standard errors from three independent assays. Values of *P < 0.05 are indicated.

We observed that overexpression of CUL1 or CUL2, but not other human Cullins, reduced the steady-state level of SAMHD1 in co-transfected 293T cells (date not shown). In an attempt to investigate whether specific Cullins are involved in HCMV-mediated SAMHD1 loss, we examined the effect of specific knockdown of CUL1, CUL2, CUL3, and CUL4 using siRNA on SAMHD1 level. HF cells were transfected with specific siRNAs for these Cullins and then infected with HCMV at an MOI of 5 for 24 or 48 h (Figure 7A). The specific effects of different siRNAs were confirmed by determining the mRNA levels of different Cullins in siRNA-transfected HF cells by qRT-PCR (Figure 7B). The level of SAMHD1 was determined by immunoblotting. In cells transfected with control siRNA (siC), this high MOI HCMV infection led to rapid loss of SAMHD1, compared to infection at an MOI of 1. We found that siRNA silencing of CUL2 and to a lesser extent CUL1 partly inhibited loss of SAMHD1 in virus-infected cells. As a control, the level of p21^CIP1^, which is degraded by the CUL4-associated CRL complex (Nishitani et al., 2008), was less effectively reduced by HCMV infection in CUL4A-depleted cells. This result did not indicate a direct role of CUL2 or CUL1 in SAMHD1 loss during HCMV infection since siCUL1 and siCUL2 transfection increased SAMHD1 level in normal HF cells and these also reduced viral late gene expression, which might be required for SAMHD1 loss (Figures 7C,D). However, the result suggests that SAMHD1 stability is regulated by CRL complexes in normal HF cells.

## Discussion

In the present study, we demonstrated that SAMHD1 depletion using shRNA increased the HCMV UL99 late gene expression by 7- to 9-fold in HF cells, confirming an antiviral role of SAMHD1 in HCMV replication. We also showed that expression of SAMHD1 is downregulated at late stages of HCMV infection at both transcription and protein levels, and that regulation at the protein level requires the CRL activity. SAMHD1 appears to restrict HCMV growth via multiple mechanisms. One study demonstrated that SAMHD1 reduces HCMV MIE gene expression in primary HF cells and THP1 cells through NF-κB downregulation at the early stage (Kim et al., 2019), while another study in MDMs suggested that SAMHD1 inhibits viral genome amplification (Businger et al., 2019). In the present study, we observed that the steady-state SAMHD1 protein level is reduced at late times of infection through a mechanism dependent on CRL complexes. Therefore, loss of SAMHD1 appears to be a viral countermeasure against SAMHD1-mediated intrinsic defense for efficient viral growth.

The CRL complex plays a key role in many cellular processes through ubiquitination and protein degradation. Modification (neddylation) of Cullins by Nedd8, a ubiquitin-like protein, is essential for formation of CRL complexes (Sarikas et al., 2011). Since the steady-state expression level of SAMHD1 was increased by expression of siRNA for CUL2 and CUL1 in HF cells (without viral gene products), SAMHD1 appears to be a normal substrate of CRL complexes. However, our observation that, unlike HCMV, UV-HCMV did not reduce SAMHD1 level strongly indicates that newly expressed viral protein(s) facilitate SAMHD1 degradation, probably indirectly facilitating SAMHD1 degradation by CUL2- or CUL1-associated CRL complexes or directly recruiting specific CRL complexes. Our analysis using a viral polymerase inhibitor suggests that viral late gene product(s) may be involved in this process. The CRL complexes are composed of a Cullin backbone, which belongs to a family of hydrophobic scaffold proteins, and its adaptors. Whether viral proteins indirectly regulate CRL complexes or directly interact with specific Cullins or its adaptors for SAMHD1 degradation is of interest and awaits further investigation. Indeed, several beta-herpesvirus proteins, such as pUL35 and pUL145 of HCMV and M27 and M35 of mouse CMV, have been shown to recruit CRL complexes to promote viral growth (for review Becker et al., 2019). However, it is unlikely that pUL145 is involved in SAMHD1 loss because HCMV Towne strain, which lacks the UL145 gene, still promoted SAMHD1 loss in our assays. Furthermore, we could not observe the effect of overexpression of these proteins on SAMHD1 level in co-transfection assays (data not shown). Recent studies also demonstrated that HCMV UL97 and MCMV M97 kinases phosphorylate SAMHD1, inactivating its antiviral activity (Businger et al., 2019; Deutschmann et al., 2019; Kim et al., 2019). Therefore, HCMV appears to have multiple countermeasures that evade restriction by SAMHD1.

Previous studies showed that formation of CRL complexes is necessary for growth of several different viruses, including HCMV (Le-Trilling et al., 2016). Consistent with these findings, when we infected HF cells with recombinant HCMV containing a reporter gene [Pol (UL54)-luciferase or pp28 (UL99)-luciferase], MLN4924 treatment led to a 4–5 log reduction of virus titers compared to control DMSO treatment and suppressed both early and late promoters (data not shown). Considering that SAMHD1 restricts the HCMV MIE gene by downregulation of NF-κB (Kim et al., 2019), the impact of MLN4924 on viral early genes may occur through SAMHD1 stabilization. It is also likely that MLN4924 may prevent the degradation of SAMHD1 and thus SAMHD1 lowers the dNTP pool, which is crucial for DNA synthesis, impairing viral late gene expression. The results of our study suggest that SAMHD1 stabilization by MLN4924 may contribute to the anti-HCMV activity of MLN4924.

## Data Availability Statement

The datasets generated for this study are available on request to the corresponding author.

## Author Contributions

SH, ML, and J-HA designed experiments, analyzed data, and wrote the manuscript. SH, ML, Y-EK, and GL performed experiments.

## Conflict of Interest

The authors declare that the research was conducted in the absence of any commercial or financial relationships that could be construed as a potential conflict of interest.
